# DAR-PCR: a new tool for efficient retrieval of unknown flanking genomic DNA

**DOI:** 10.1186/s13568-022-01471-1

**Published:** 2022-10-12

**Authors:** Tianyi Sun, Mengya Jia, Lingqin Wang, Zhaoqin Li, Zhiyu Lin, Cheng Wei, Jinfeng Pei, Haixing Li

**Affiliations:** 1 Key Laboratory of Poyang Lake Environment and Resource Utilization of Ministry of Education, School of Environmental and Chemical Engineering, Nanchang University, 330031 Nanchang, PR China; 2 State Key Laboratory of Food Science and Technology, Nanchang University, 330047 Nanchang, PR China; 3 Sino-German Joint Research Institute, Nanchang University, 330047 Nanchang, PR China; 4 Charles W. Davidson College of Engineering, San Jose State University, 95192 San Jose, CA USA

**Keywords:** Genome walking, Differential annealing, Intra-strand annealing, Racket-like DNA, Primer root, Primer bud

## Abstract

Various PCR-based genome-walking methods have been developed to acquire unknown flanking DNA sequences. However, the specificity and efficacy levels, and the operational processes, of the available methods are unsatisfactory. This work proposes a novel walking approach, termed differential annealing-mediated racket PCR (DAR-PCR). The key to DAR-PCR is the use of primer-mediated intra-strand annealing (ISA). An ISA primer consists of a 5’ root homologous to the known sequence and a heterologous 3’ bud. In the single low-stringency cycle, the ISA primer anneals to a site on an unknown region and extends towards the sequence-specific primer (SSP) 1 site, thereby forming a target single-stranded DNA bound by the SSP1 complement and the ISA primer. In the subsequent more stringent cycles, its complementary strand is accumulated, owing to the differential annealing between the moderate-stringency ISA primer and the high-stringency SSP1. The accumulation of this strand provides an opportunity for ISA mediated by the ISA primer root. A loop-back extension subsequent to ISA occurs, creating a racket-like DNA with the known region positioned at both ends of the unknown sequence. This DNA is exponentially amplified during the secondary PCR driven by an SSP pair inner to SSP1. DAR-PCR was validated as an efficient walking method by determining unknown flanking sequences in *Lactobacillus brevis* and *Oryza sativa*.

**Supplementary Information:**

The online version contains supplementary material available at 10.1186/s13568-022-01471-1.

## Introduction

Genome walking refers to a sequence-dependent strategy used to access unknown sequences flanking known DNA regions. The genomic DNA library-based walking technique is unpopular owing to the heavy workload and high cost. PCR-based approaches have been favored because of their efficiency, rapidity and simplicity (Rishi et al. 2004). To date, numerous PCR-based methods have been developed and widely applied to acquire unknown flanking sequences (Kotik 2009; Kim et al. 2021a, b). These PCR methods differ largely in experimental processes but can be classified into three categories according to their underlying principles: (i) inverse PCR (Ochman et al. 1988; Benkel and Fong 1996; Uchiyama and Watanabe 2006); (ii) terminal modification-dependent PCR (Tsuchiya et al. 2009; Ashrafmansouri et al. 2020); and (iii) randomly primed PCR (Liu and Whittier 1995; Tan et al. 2005; Wang et al. 2013; Zhang et al. 2018).

Inverse PCR requires the endonuclease digestion of genomic DNA and the subsequent self-cyclization of the digested DNA. It thus produces cyclized DNA in which unidentified upstream and downstream regions are situated adjacent to each other, with the known DNA being placed on both ends of this unknown hybrid (Triglia et al. 1988; Wang et al. 2021). This cyclized DNA serves as a template for PCR using two sequence-specific primers (SSPs) having opposite orientations. The two primers extend outward from the known region of this special template to amplify unknown flanking segments. Inverse PCR features high specificity because the primers used are completely sequence-specific (Tsaftaris et al. 2010; Trinh et al. 2014). However, the efficiency of inverse PCR is relatively low. In addition, extra operations prior to PCR amplification make this method complex (Uchiyama and Watanabe 2006).

Terminal modification-dependent PCR requires the endonuclease digestion of the genome, followed by the ligation of the digested DNA fragments to a synthetic oligonucleotide (Siebert et al. 1995; Ishihara et al. 2017). A ligated target product is enriched by two to three rounds of PCRs performed using the oligonucleotide primer successively paired with nested SSPs (Tsuchiya et al. 2009; Reddy et al. 2012). Clearly, the elimination of the non-specific background arising from the oligonucleotide primer is an issue in this strategy (Alquezar-Planas et al. 2020). Although improvements in the oligonucleotide, such as the dephosphorylation of the 5’ end or amination of the 3’ end, have been made to enhance specificity, non-specific amplification has not yet been effectively overcome (Tsuchiya et al. 2009; Bae and Sohn 2010; Ashrafmansouri et al. 2020).

Randomly primed PCR is a pretreatment-free DNA-walking approach (Jia et al. 2017). A single low-stringency cycle allows the walking primer to arbitrarily anneal to genomic DNA and prime DNA polymerization. As a result, a pool of DNA fragments are produced (Li et al. 2015; Chang et al. 2018). The target DNA becomes major product after two to three rounds of PCRs are conducted using the walking primer successively paired with nested SSPs (Zhou et al. 2012). Thermal asymmetric interlaced PCR (Liu and Whittier 1995), universal fast walking (Myrick and Gelbart 2002) and its variants (Park 2005; Wang et al. 2007), and partially overlapping primer-based PCR (Li et al. 2015) and its improved versions (Chang et al. 2018; Wang et al. 2022), are types of randomly primed PCR. Nevertheless, for thermal asymmetric interlaced PCR, non-target DNAs arising from the walking primer are inevitable, as one in three cycles must be of low stringency. The other randomly primed PCRs involve complicated operations or require several walking primers (Thirulogachandar et al. 2011; Tan et al. 2019).

In this work, we describe differential annealing-mediated racket PCR (DAR-PCR), an efficient tool for genome walking. This method relies on intra-strand annealing (ISA) at an ISA locus and a subsequent loop-back extension along the known region. As a result, a racket-like DNA is synthesized with the known region being incorporated on each side of the unknown DNA. This racket-like DNA serves as template in the subsequent nested PCR. For a proof-of-concept, DAR-PCR was successfully employed to determine the sequences of the unknown regions flanking the *Lactobacillus brevis* CD0817 glutamate decarboxylase gene (*gadA*) and *Oryza sativa* hygromycin gene (*hyg*).

## Materials and methods

### Extraction of genomic DNAs

Genomic DNA of *L. brevis* CD0817 (= CCTCCM2018462) was extracted using the Bacterial Genomic DNA Isolation Kit (Tiangen Biotech Co., Ltd, Beijing, China) in accordance with the manufacturer’s instructions. *Oryza sativa* genomic DNA was kindly provided by the Peng laboratory at Nanchang University (Nanchang, China).

### Oligonucleotides

An ISA primer contains a sequence-specific 5’ root appended to a random 3’ bud. The root is fixed and responsible for ISA. The bud consists of very few (here, 0 to 2) nucleotides heterologous to the known sequence. Therefore, one ISA root can be used in many ISA primers. All the ISA primers are 15–20 bp and have a moderate melting temperature (Tm) of 45–55℃. The SSPs were derived from the *gadA* locus (CP032931.1) and *hyg* gene (KF206149.1), and have a high Tm values of 60–65℃. The software Oligo 7 (Molecular Biology Insights, Inc., USA) was used to evaluate primer Tm and potential primer-dimer or hairpin formation. Any primer or primer pair should not form an obvious dimer or hairpin (Table 1).

**Table 1:** Primers used in this study

Primary PCR			Secondary PCR
**ISA primer**		**SSP1**	**Inner SSP pair (SSP2/SSP3)**
*gad*-ISA1	**TGAAAACTAACCGGCTT**AC	*gad*-1: TCCATACCCTCATCTCCATTTCCAT	*gad*-2: AACTATCACCCCACAACGTCATCTC *gad*-3: ACCGTTCATAGGCGAAATTGTTTGT
*gad*-ISA2	**TAAACCTGCGTAAAAA**CT		
*gad*-ISA3	**AACCGGCTTTTTAAACT**		
*hyg*U-ISA1	**CGGGCGTACACAAATC**TC	*hyg*U-1: GGCGTATATGCTCCGCATTGGTCTT	*hyg*U-2: CGGCAATTTCGATGATGCAGCTTGG *hyg*U-3: GACCGATGGCTGTGTAGAAGTACTC
*hyg*U-ISA2	**GCAATCGTCCGATCC**CT		
*hyg*U-ISA3	**AAATCGCCCGCAGAA**		
*hyg*D-ISA1^§^	**CTAAACTCCCCAATGTC**	*hyg*D-1: GCCATGTAGTGTATTGACCGATTCC	*hyg*D-2: CAGTTCGGTTTCAGGCAGGT *hyg*D-3: CATATCCACGCCCTCCTACA
*hyg*D-ISA1a^§^	**CTAAACTCCCCAATGTC**T		
*hyg*D-ISA1b^§^	**CTAAACTCCCCAATGTC**C		
*hyg*D-ISA1c^§^	**CTAAACTCCCCAATGTC**TC		
*hyg*D-ISA1d^§^	**CTAAACTCCCCAATGTC**CA		
*hyg*D-ISA2	**AGTGCCGATAAACATAA**		

Note: ISA roots are underlined. Buds are unmarked nucleotides located at the 3’ ends of ISA roots. ISA primers paired with SSP1 in the same row were used in the primary PCR; corresponding secondary PCRs were performed using the inner SSP pair (SSP2/SSP3) in the same row. ^§^ indicates ISA primers derived from the same ISA root

### PCR system and thermal cycling

The DAR-PCR consists of two rounds of nested PCR reactions. Genomic DNA was used as the template of the primary PCR using SSP1 and an ISA primer. The 50-µL primary PCR mixture included 0.4 mM of each dNTP, 0.2 µM of each primer, genomic DNA (10–100 ng for the microbe and 100–1,000 ng for *Oryza sativa*), 1× LA PCR buffer II (Mg^2+^ plus) and 2.5 U of TaKaRa LA Taq. In total, 1 µL of primary PCR product was used as the template in the 50-µL secondary PCR reaction, along with two inner SSPs instead of the primary PCR primers. The other components of secondary PCR were identical to those of the primary PCR.

The primary PCR included the following four stages: (i) five slightly high-stringency (60℃) cycles (SHSC); (ii) one low-stringency (25℃) cycle (LSC); (iii) 15 moderate-stringency (55℃) cycles (MSC); and (iv) 25 high-stringency (65℃) cycles (HSC). Secondary PCR was composed of 35 SHSCs. The detailed thermal cycling parameters are presented in Table 2.

**Table 2:** Thermal cycling parameters for the DAR-PCR method

Round of PCR	Stage	Thermal conditions	Cycle number
Primary		94 °C, 2 min	
	1	94 °C 30 s, 60 °C 30 s, 72 °C 2 min	5
	2	94 °C 30 s, 25 °C 30 s, 72 °C 2 min	1
	3	94 °C 30 s, 55 °C 30 s, 72 °C 2 min	15
	4	94 °C 30 s, 65 °C 30 s, 72 °C 2 min	25
		72 °C 5 min	
1 µL of the product was directly used as template for secondary PCR			
Secondary		94 °C, 2 min	
	1	94 °C 30 s, 60 °C 30 s, 72 °C 2 min	35
		72 °C 5 min	

### DNA sequencing and analysis

PCR products were electrophoresed on 1% agarose gels and stained with ethidium bromide to obtain visible DNA bands. The clear DNA bands were recovered using an Agarose Gel DNA Purification Kit Version 2.0 (TaKaRa, Beijing, China) and were entrusted to Sangon Biotech Co., Ltd. (Shanghai, China) for sequencing.

## Results

### Outline of DAR-PCR

**Fig. 1:**
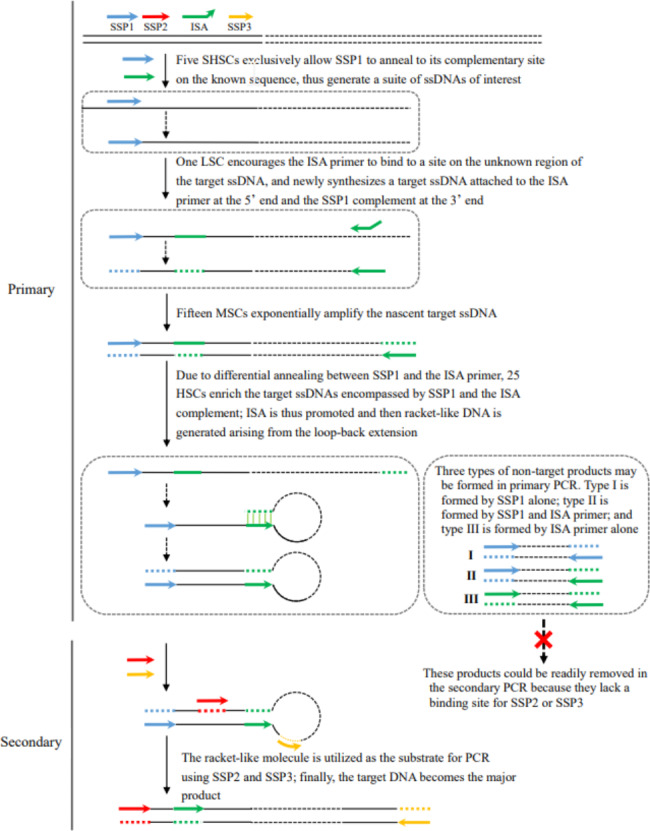
Schematic depiction of DAR-PCR. Primary PCR is performed using SSP1 and the ISA primer; and the secondary PCR is performed using SSP2 and SSP3. The bud heterologous to the known DNA is indicated by the up-ended arrow. The solid lines denote known sequences; Dotted lines denote unknown sequences. SHSC: slightly high-stringency (60℃) cycle; LSC: low-stringency (25℃) cycle; MSC: moderate-stringency (55℃) cycle; HSC: high-stringency (65℃) cycle. The PCR primers are indicated by numbered arrows, and their locations in relation to the relevant strand of genomic DNA are shown on top of the diagram in Step 1

The principle and process of DAR-PCR are shown in Fig. 1. The key to this method is the design and application of the ISA primer. As described in the Materials and Methods section, an ISA primer contains a sequence-specific root with a mismatched bud attached at the 3’ end. For primary PCR, the initial five SHSCs (60℃) only allow SSP1 (Tm 60–65℃) to bind its complementary site within the known sequence and elongate towards the unknown region, thereby exclusively increasing the copies of the target single-stranded DNA (ssDNA). The following one LSC (25℃) permits the ISA primer (Tm 45–55℃) to arbitrarily anneal to some position on the unknown flank and to prime DNA polymerization towards the known region, producing a molecule enclosed by the ISA primer and SSP1. This new molecule is exponentially amplified in the following 15 MSCs (55℃). The strand of this new molecule, with SSP1 at the 5’ end and the ISA complement at the 3’ end, is preferentially amplified in the next 25 HSCs (65℃) owing to the differential annealing of SSP1 and the ISA primer. In addition, some of the strands undergo ISA at the ISA locus, and thereafter, a racket-like DNA is synthesized using the protruding 5’ part as the template. As a result, the known region between SSP1 and ISA is incorporated into each side of the unknown segment. The racket-like DNA can then be used as the template in the secondary PCR to identify the unknown region.

The secondary PCR, which is performed using two SSPs (SSP2 and SSP3) inner to SSP1, is a type of classical end-to-end PCR. The positional relationship of SSP2 and SSP3 avoids the production of an overlap at the two ends of the final PCR product; consequently, exponential amplification is achieved. Additionally, any non-target product generated in the primary PCR is eliminated owing to the lack of a perfect binding-site for SSP2 or SSP3.

### Validation of DAR-PCR

**Fig. 2:**
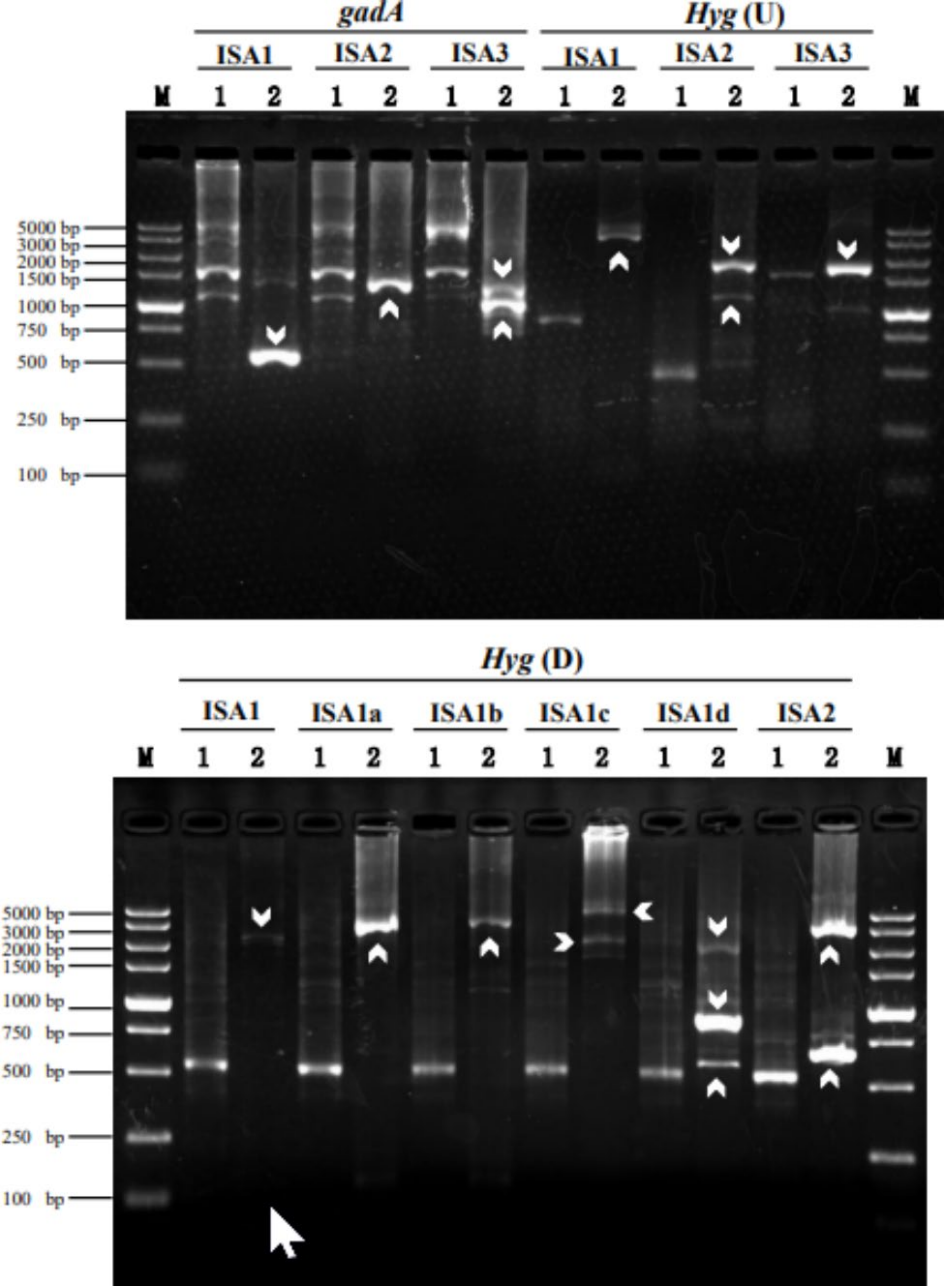
Walking upstream of *gadA*(U) and *hyg* (U), as well as downstream of *hyg* (D). The ISA primers listed under each gene were respectively paired with SSP1 during the primary PCR reactions; the corresponding secondary PCRs were then performed using SSP2 and SSP3 (as described in Table 1). Lane 1: primary PCR; lane 2: secondary PCR; white arrows indicate target bands; and M: DL5000 DNA marker

The feasibility of DAR-PCR was tested by probing unknown regions flanking the *L. brevis* CD0817 *gadA* gene and the *Oryza sativa hyg* gene. As illustrated in Fig. 2, more than one clear band appeared in all the secondary PCR reactions. Sequencing data demonstrated that all the dominant bands were target products, verifying the high specificity of the current method (supplementary materials Fig. S1-S3). The longest DNA fragments obtained in each walking experiment ranged from 1.5 to 5.0 kb (Fig. 2), indicating the high efficiency of DAR-PCR.

## Discussion

PCR-based genome-walking strategies have been unsuccessful owing to non-specific amplification attributed to walking primers (Tonooka and Fujishima 2009). In general, improvements to the existing PCR-based techniques have been aimed at controlling the balance between specificity and efficiency (Myrick and Gelbart 2002; Kim et al. 2021a, b). For DAR-PCR, however, this is unnecessary because its secondary PCR involves only site-specific amplification. The current technique possesses high efficiency and specificity that are equal to those of classical end-to-end PCR.

Traditional panhandle PCR (Jones and Winistorfer 1992), inverse PCR (Ochman et al. 1988; Benkel and Fong 1996; Uchiyama and Watanabe 2006) and terminal modification-dependent PCR (Tsuchiya et al. 2009; Ashrafmansouri et al. 2020) involve pretreatments prior to the PCR reactions, such as endonuclease cleavage and DNA ligation, which reduce the walking efficiency and increase the cost and workload (Jeung et al. 2005). Therefore, the development of a truly PCR-based genome-walking technique is desired. Universal fast walking (Myrick and Gelbart 2002) and its variants (Park 2005; Wang et al. 2007) are completely PCR-based techniques. However, these techniques do not always result in positive outcomes because the exclusive single walking primer sometimes fails to bind to the DNA of interest. Moreover, the number of ssDNAs anticipated to form panhandle-like molecules is limited, which also reduces the success rates of these methods. In our method, more than one ISA root can be obtained from the known region between SSP2 and SSP3 (Fig. 1), and any ISA root can result in many ISA primers by adding buds at the 3’ end. Thus, SSP1 can pair with various ISA primers, allowing a set of parallel PCR reactions to be conducted. We hypothesize that at least one ISA primer will successfully anneal to some site on the unknown DNA of interest at the low-stringency cycle, resulting in the guaranteed success of the method. In addition, an ISA primer should have a distinctive annealing site because of its unique 3’ bud. Thus, a rather long fragment may be produced if parallel PCR reactions are performed. These features ensure the high walking efficiency and success rate of DAR-PCR.

TAIL-PCR (Tan et al. 2019) and POP-PCR (Li et al. 2015) are versatile genome-walking methods. The two methods dilute undesired products owing to the differential annealing between the walking primer and SSP. Thus, the two methods enrich target DNAs by having the efficiency of the specific amplification surpass that of non-specific amplification, which implies that non-specific amplification is not negligible. For specificity and efficiency, DAR-PCR is superior to TAIL-PCR or POP-PCR because its secondary reaction is performed using a completely sequence-specific primer pair.

In some cases, multiple bands appeared in the gel (Fig. 2). This multi-band phenomenon is common in most PCR-based DNA-walking technologies, and it may be interpreted as the walking primer annealing to multiple sites on the unknown region of interest (Tan et al. 2019; Liu and Chen 2007).

A new tool, DAR-PCR, has been established for the efficient determination of unknown DNA. This method dispenses with extra steps prior to PCR reactions and decreases the number of artifacts that occur in available genome-walking strategies. This method has many potential applications in molecular biology and related areas. DAR-PCR is a promising alternative to the existing DNA walking methods owing to its high specificity and efficiency, along with its simplicity.

## Electronic supplementary material

Below is the link to the electronic supplementary material.

Supplementary Material 1: Figure S1. Alignment of confirmatory sequencing data and locations of primers used to probe upstream of *gadA*. Figure S2. Alignment of confirmatory sequencing data and locations of primers used to probe upstream of *hyg*. Figure S3. Alignment of confirmatory sequencing data and locations of primers used to probe downstream of *hyg*.

## Data Availability

The datasets supporting the conclusions of this article are included within the article.

## Abbreviations

DAR-PCR: Differential annealing-mediated racket PCR.
*gadA*: Glutamate decarboxylase gene A.
*hyg*: Hygromycin gene.
ISA: Intra-strand annealing.
PCR: Polymerase chain reaction.
ssDNA: Single-stranded DNA.
SSP: Sequence-specific primer.
Tm: Melting temperature.
SHSC: Slightly high-stringency cycle.
LSC: low-stringency cycle.
MSC: Moderate-stringency cycle.
HSC: High-stringency cycle.
