# Supplementary material for: DAR-PCR: a new tool for efficient retrieval of unknown flanking genomic DNA
Source: AMB Express. 2022 Oct 12;12:131. doi: 10.1186/s13568-022-01471-1 (PMC9556680; doi:10.1186/s13568-022-01471-1)

**Figure S1.** Alignment of confirmatory sequencing data and locations of primers used to probe upstream of *gadA*. The primer regions are underlined, and are denoted using the same primer names as in Table 1.


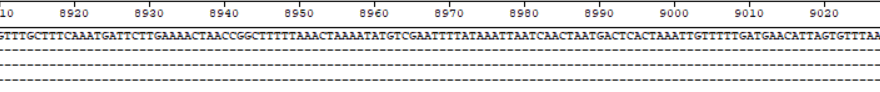

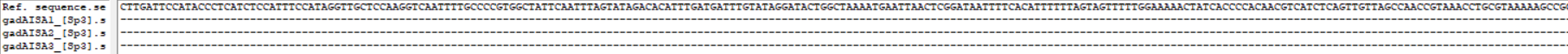

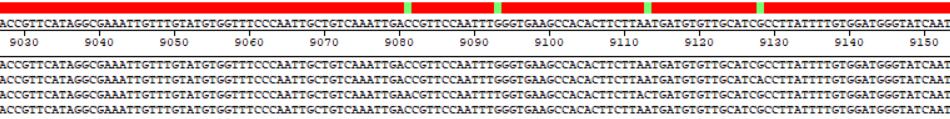

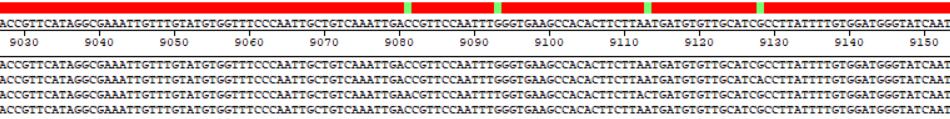


***gad*-1**

***gad*-2**

***gad*-3**

***gad*-ISA1**

**Known sequence**

**Unknown sequence**

***gad*-ISA3**

***gad*-ISA2**

**Figure S2.** Alignment of confirmatory sequencing data and locations of primers used to probe upstream of *hyg*. The primer regions are underlined, and are denoted using the same primer names as in Table 1.


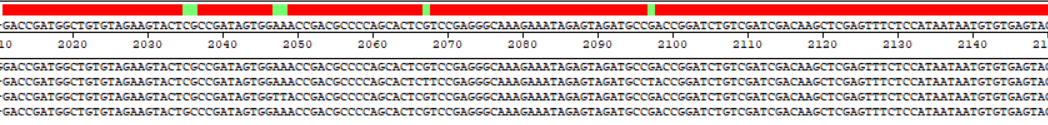

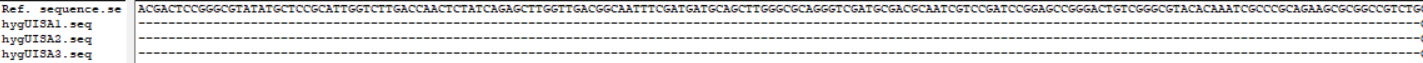


**Known sequence**

**Unknown sequence**

*hyg*U-3

*hyg*U-1

*hyg*U-2

*hyg*U-ISA2

*hyg*U-ISA1

*hyg*U-ISA3

**Figure S3.** Alignment of confirmatory sequencing data and locations of primers used to probe downstream of *hyg*. The primer regions are underlined, and are denoted using the same primer names as in Table 1.


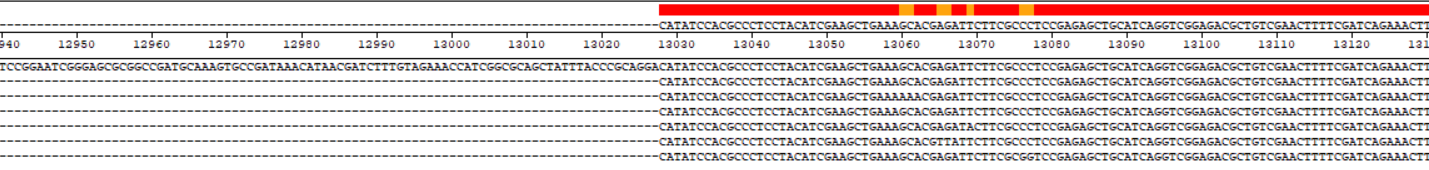


*hyg*U-ISA1d

*hyg*U-ISA1c

*hyg*U-ISA1b

*hyg*U-ISA1a

*hyg*U-ISA1


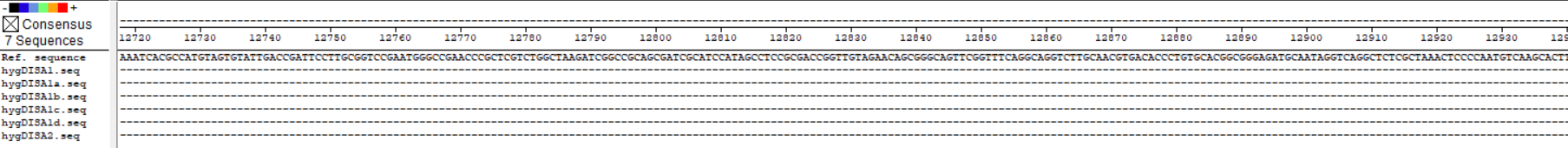

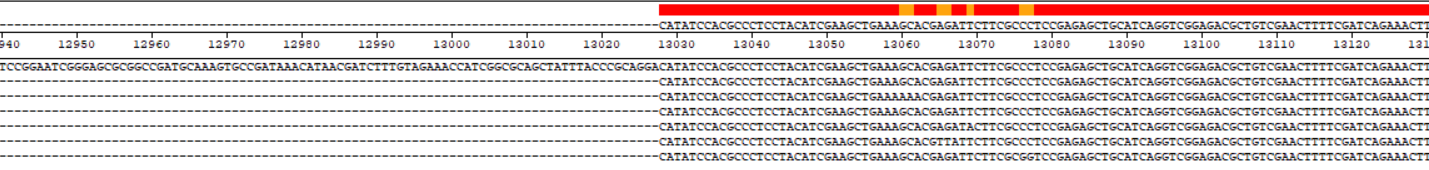


**Known sequence**

**Unknown sequence**

*hyg*D-3

*hyg*D-1

*hyg*D-2

*hyg*D-ISA2


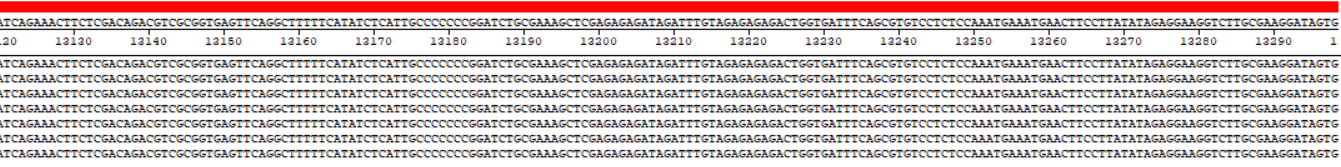

Supplement: Supplementary file 1 — Supplementary Material 1: Figure S1. Alignment of confirmatory sequencing data and locations of primers used to probe upstream of gadA. Figure S2. Alignment of confirmatory sequencing data and locations of primers used to probe upstream of hyg. Figure S3. Alignment of confirmatory sequencing data and locations of primers used to probe downstream of hyg. [file 13568_2022_1471_MOESM1_ESM.docx]
